# Radiation‐Hardened Perovskite Solar Cells Enabled by Redox‐Active V_2_O*_x_* Hole Transport Layer for Space Applications

**DOI:** 10.1002/smsc.70304

**Published:** 2026-05-28

**Authors:** EQ Han, Su‐Ho Ahn, Eunyoung Choi, Su‐Min Lee, Jaeho Lee, Bo Wei Zhang, Miaoqiang Lyu, Jung‐Ho Yun

**Affiliations:** ^1^ Air & Environment Energy Nexus Lab, Department of Environmental Science and Engineering, College of Engineering Kyung Hee University Gyeonggi‐do Republic of Korea; ^2^ School of Chemical Engineering The University of Queensland Brisbane Australia

**Keywords:** perovskite solar cells, radiation, redox‐buffer, space, vanadium oxide

## Abstract

Recent advances in space missions have motivated increased research into photovoltaic technologies with improved tolerance to radiation environments in space. Here, we evaluate proton radiation resilience in perovskite solar cells via an employment of a vanadium (V) oxide (V_2_O*_x_*)/self‐assembled layer (SAM) bilayer hole transport layers (HTLs). We show that the V_2_O*_x_*/SAM bi‐HTL initially promotes more efficient charge extraction, as evidenced by enhanced photoluminescence quenching, while higher proton irradiation doses progressively weaken this benefit, consistent with the irradiation‐driven interfacial modification rather than significant absorber degradation. Notably, X‐ray photoelectron spectroscopy reveals irradiation‐induced reduction of V_2_O*_x_*, with oxygen vacancy formation and increased V^4+^ content, indicating a redox‐active interface that promotes hole extraction while buffering proton‐induced interfacial degradation. In addition, analysis of the Pb core level reveals that SAM‐only devices exhibit the emergence and growth of metallic Pb species after proton irradiation, whereas devices incorporating V_2_O*_x_* act as a redox buffer that suppress metallic Pb formation across proton irradiations. This work first demonstrates the effects of metal oxide charge transport layer engineering on radiation‐hardened perovskite interfaces and advances the development of durable, lightweight photovoltaic technologies for space power applications.

## Graphical abstract

A redox‐active V_2_O*_x_*/self‐assembled layer bilayer hole transport layer improves the proton radiation resilience of perovskite solar cells for space applications. The V_2_O*_x_* interface suppresses irradiation‐induced metallic Pb formation while buffering interfacial degradation, highlighting metal oxide interfacial engineering as a practical route toward durable lightweight space photovoltaics.

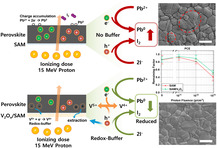

## 1 Introduction

Despite rapid progress in device efficiency over 27%, the development of stable and radiation‐resilient organic‐based charge transport layers (CTLs) remains a key bottleneck for space deployment [1, 2, 3]. In low earth orbit (LEO), photovoltaic devices are exposed to a complex radiation environment dominated by energetic protons and electrons, which induce both ionizing energy loss (IEL) and nonionizing energy loss (NIEL) [4, 5]. While NIEL is primarily responsible for displacement damage dose, vacancy formation, and the generation of interfacial trap states that degrade long‐term charge extraction, IEL causes total ionizing dose (TID) effects, including persistent charge accumulation, bond scission, and radiation‐induced chemical reactions at organic–inorganic interfaces [6, 7, 8]. Importantly, the relative contribution of IEL and NIEL strongly depends on proton energy. Lower‐energy protons deposit a larger fraction of their energy locally within the active layers and are therefore more effective in inducing displacement damage‐dominated degradation, whereas higher‐energy protons penetrate more deeply through the device stack and place greater emphasis on ionization‐related effects over a broader spatial range [4, 7, 9, 10]. Accordingly, proton irradiation studies at different energies do not probe the same degradation regime and should be interpreted with care when comparing radiation‐hardness results. These IEL‐driven processes can be detrimental to organic hole transport layers (HTLs) such as self‐assembled monolayers (SAMs) and PEDOT:PSS, which rely on *π*‐conjugated molecular backbones and weak intermolecular bonding [11, 12, 13]. Under prolonged ionizing radiation, these materials are prone to photochemical degradation, chain scission, and interfacial reactions that modify dipole alignment and energy‐level matching, leading to irreversible performance loss [1, 11, 12]. Importantly, such degradation pathways cannot be fully accounted for by NIEL‐based displacement damage models alone, underscoring the vulnerability of organic CTLs when exposed to the combined IEL–NIEL radiation environment in LEO [14, 15].

These limitations motivate a shift toward inorganic CTLs with higher thermal and chemical hardness and potentially reduced susceptibility to radiation‐driven electronic and chemical decomposition. Gamma radiation (*γ*‐radiation) has been reported to modify the oxygen‐related defects and electrical properties in SnO_2_ [16, 17]. This ionizing radiation predominantly results in oxide trapped charge build‐up and interface‐state generation, while transition metal oxide CTLs in photovoltaics function as electronically active, defect‐mediated semiconductors. Their transport and interfacial energetics depend on native point defects and mixed‐valence chemistry, implying that radiation‐induced defect formation can shift redox equilibrium and band alignment rather than acting solely as an irreversible trap generation process. V_2_O*_x_* is particularly compelling because it can provide both efficient hole extraction and strong electron blocking through deep valence band alignment and optical transparency [18, 19, 20, 21]. Importantly, the radiation resilience of V_2_O*_x_*‐based HTLs may depend not only on stoichiometric band alignment but also on defect chemistry. Thermally evaporated V_2_O*_x_* films inherently tend to form oxygen‐deficient V_2_O*_x_* (*x* ≤ 5), since vacuum deposition conditions favor partial oxygen loss and stabilization of oxygen vacancy‐rich substoichiometric phases, which can increase electrical conductivity and modify interfacial energetics [19, 22, 23, 24]. Such preexisting defect states may act as an electronic buffer that accommodates additional radiation‐induced point defects and mitigates significant loss of hole extraction, yet this hypothesis remains experimentally underexplored for HTLs in inverted perovskite solar cells. There have been previous reports on gamma ray‐irradiated perovskite solar cells (n–i–p structure) incorporating vanadium oxide‐containing CTLs, where the effects of gamma rays on recombination dynamics, defect concentration, and device performance were examined [25, 26]. However, these studies did not explicitly isolate the independent mechanistic role of vanadium oxide in radiation hardening for perovskite solar cells.

In this work, we investigate thermally evaporated vanadium oxide as a radiation‐resilient and multifunctional HTL for inverted perovskite solar cells for space applications. To distinguish our objective from low‐energy proton studies that mainly emphasize displacement damage in the absorber, we selected a 15 MeV proton condition to probe a more penetrating irradiation regime, where ionization‐related interfacial damage and defect activation in the full device stack become more relevant. We evaluate device‐level hardness under proton irradiation by tracking performance evolution as a function of accumulated fluence. Finally, we correlate postirradiation photovoltaic behavior with changes in V_2_O*_x_* oxidation state and interface chemistry to test whether oxygen vacancy‐mediated defect compensation can suppress radiation‐induced interfacial degradation, providing design guidance for oxide HTLs enabling reliable, high‐specific‐power perovskite photovoltaics in LEO environments.

## 2 Experimental Section

### 2.1 Materials

*Chemicals*: *N*, *N*‐dimethylformamide (DMF, anhydrous) and dimethyl sulfoxide (DMSO, anhydrous), ethanol (anhydrous), isopropanol (IPA, anhydrous), anisole (ANS, anhydrous), fullerene (C_60_), bathocuproine (BCP) and phenethylammonium iodide (PEAI), cesium iodide (CsI), and vanadium (V) oxide (V_2_O_5_) were purchased from Sigma‐Aldrich. Lead iodide (PbI_2_) and [2‐(3,6‐dimethoxy‐9H‐carbazol‐9‐yl)ethyl]phosphonic acid (MeO‐2PACz) were purchased from the Tokyo Chemical Industry (TCI). Formamidine iodide (FAI) was purchased from GreatCell Solar Materials.

### 2.2 Preparation of V_2_O_x_

V_2_O*_x_* films were deposited on indium‐doped tin oxide (ITO) glass substrates by thermal evaporation (Korea Kiyon, South Korea). V_2_O_5_ powder (TASCO, USA) was loaded into an alumina (Al_2_O_3_) crucible and evaporated in a high‐vacuum chamber (base pressure ≈10^−6^ Torr). The deposition rate was maintained at 0.1 Å/s, and the film thickness was set to 10 nm. After deposition, the samples were kept in the vacuum chamber to avoid rapid cooling and were then vented by slowly backfilling with N_2_ before unloading. The as‐deposited V_2_O*_x_* films exhibited a yellowish appearance. For postannealing, the films were annealed at 150°C for 5 min either in an N_2_‐filled glove box or under ambient conditions. After annealing, the films became more transparent with a reduced yellow color.

### 2.3 Device Fabrication

Transparent conductive oxide (TCO) glass substrates (indium‐doped tin oxide (ITO) or fluorine‐doped tin oxide (FTO)) were cleaned by sequential sonication in acetone, ethanol, and 2‐propanol for 15 min each, respectively. The substrates were dried in an oven at 60°C for 30 min and followed by UV treatment for 15 mins to remove residual organic contaminants and increase wettability. The SAM (MeO‐2PACz) was dissolved in anhydrous ethanol at 0.5 mg/mL. For preparing SAM‐only samples, 100 μL of SAM solution was spin‐coated on TCO substrate at 100 rpm for 10 s and 3000 rpm for 30 s, followed by annealing on a hotplate at 100°C for 10 min. For V_2_O*_x_*/SAM samples, V_2_O*_x_* was firstly deposited on TCO substrates and then the SAM was deposited using the same spin‐coating and annealing procedure. A 1.5 M of Cs_0.05_FA_0.95_PbI_3_ perovskite precursor was dissolved in mixed solvent (DMF/DMSO, 4:1 vol%) and stirred for 1 h. Then, 100 μL of perovskite precursor solution was spin‐coated onto SAM or V_2_O*_x_*/SAM‐deposited substrates at 4000 rpm for 22 s with an acceleration of 800 rpm/s. Three hundred μL of ANS was cast 5 s before the end of the spin‐coating step. The films were annealed at 100°C for 1 h and then cooled to room temperature. Subsequently, PEAI (2 mg/mL in IPA) layer was deposited and then annealed at 100°C for 10 min. A C_60_ electron transport layer (20 nm) and BCP buffer layer (6 nm) were deposited via thermal evaporation at 0.1 Å/s. Finally, 100 nm of Ag electrode was thermally evaporated through a shadow mask to define the device area.

### 2.4 Proton Irradiation

Proton beam irradiation was performed at the Korea Multi‐purpose Accelerator Complex (KOMAC, Gyeongju, South Korea) of the Korea Atomic Energy Research Institute (KAERI), using TR23 as the proton beam source. The fabricated devices were exposed to 15 MeV protons at total fluences of 10^11^–10^13^ p/cm^2^. During irradiation, the facility conditions were maintained at room temperature and a relative humidity of ≈50%. After irradiation, the samples were stored in a humidity‐controlled desiccator at KOMAC between subsequent characterizations.

### 2.5 Characterization

The photocurrent density–photovoltage curves (*J–V* curves) of devices were recorded under AM 1.5G illumination at an intensity of 100 mW/cm^2^ (Ossila and Newport). A metal aperture (0.096 cm^2^) was superimposed on the device to define the active area during the measurement. Surface images of perovskite using scanning electron microscopy (SEM, Gemini360, Carl Zeiss) with 15 kV. Five nm of Pt was coated before SEM measurement. The X‐ray diffraction (XRD) measurement was performed on D8 Advance (Bruker) with cobalt K*α* radiation, and the result was further converted into that of the copper target. The steady‐state photoluminescence (PL) emissions from samples of perovskite only and samples of ETL/perovskite were measured at 450 nm light sources excitation using a monochromatized Xe lamp. UV–Vis absorption spectra were obtained with a JASCO‐670 spectrophotometer. The X‐ray photoelectron spectroscopy (XPS) spectra were measured by a Kratos Axis Supra Plus XPS with an Al K*α* radiation source, and all the spectra were calibrated by using C 1s (284.8 eV) as a reference.

## 3 Results and Discussion

### 3.1 Characterizations of Thermally Evaporated V_2_O_x_

**FIGURE 1:**
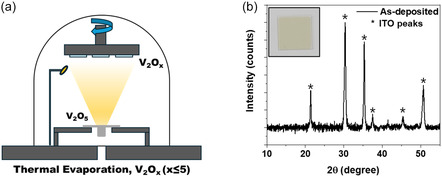
Thermally evaporated V_2_O*_x_* film characterization. (a) Illustration of thermal evaporation system for the V_2_O*_x_* layer, and (b) XRD pattern of the as‐deposited V_2_O*_x_* film, confirming an amorphous phase. (* markers in the figures indicate ITO peaks (211), (222), (400), (411), (431), and (440) from left to right.).

In solar cells, amorphous V_2_O*_x_* has been widely reported to be more suitable as a HTL than its crystalline counterpart, owing to its uniform electronic structure, reduced interfacial recombination, and compatibility with low‐temperature processing [24, 27, 28]. In this study, V_2_O*_x_* films were fabricated by thermal evaporation (Figure 1a). XRD measurements were performed to examine the structural nature and crystallinity of the thermally evaporated V_2_O_*x*_ films (Figure 1b). The as‐deposited films exhibit no specific crystalline peaks, confirming their amorphous nature. The amorphous structure was preserved even after postannealing treatment (150°C for 5  min) conducted in an N_2_‐filled glove box and in ambient condition, indicating excellent structural stability (Figure S1a–b). The postannealed film samples became slightly brighter, as shown in the inlet image of Figure S1a–b, consistent with previously reported [29]. However, XRD results confirmed that the films remained amorphous phase under the tested annealing conditions. Furthermore, UV–visible absorption spectroscopy was performed to evaluate the optical impact of the V_2_O*_x_* interlayer beneath the perovskite film (Figure S2). The as‐deposited V_2_O*_x_* exhibited higher absorption in the visible range, which could reduce the photon flux reaching the perovskite; therefore, a brief postannealing step was adopted to minimize parasitic absorption prior to device fabrication. These results demonstrate that thermally evaporated V_2_O*_x_* films maintain an amorphous structure suitable for application as HTLs in perovskite solar cells.

**FIGURE 2:**
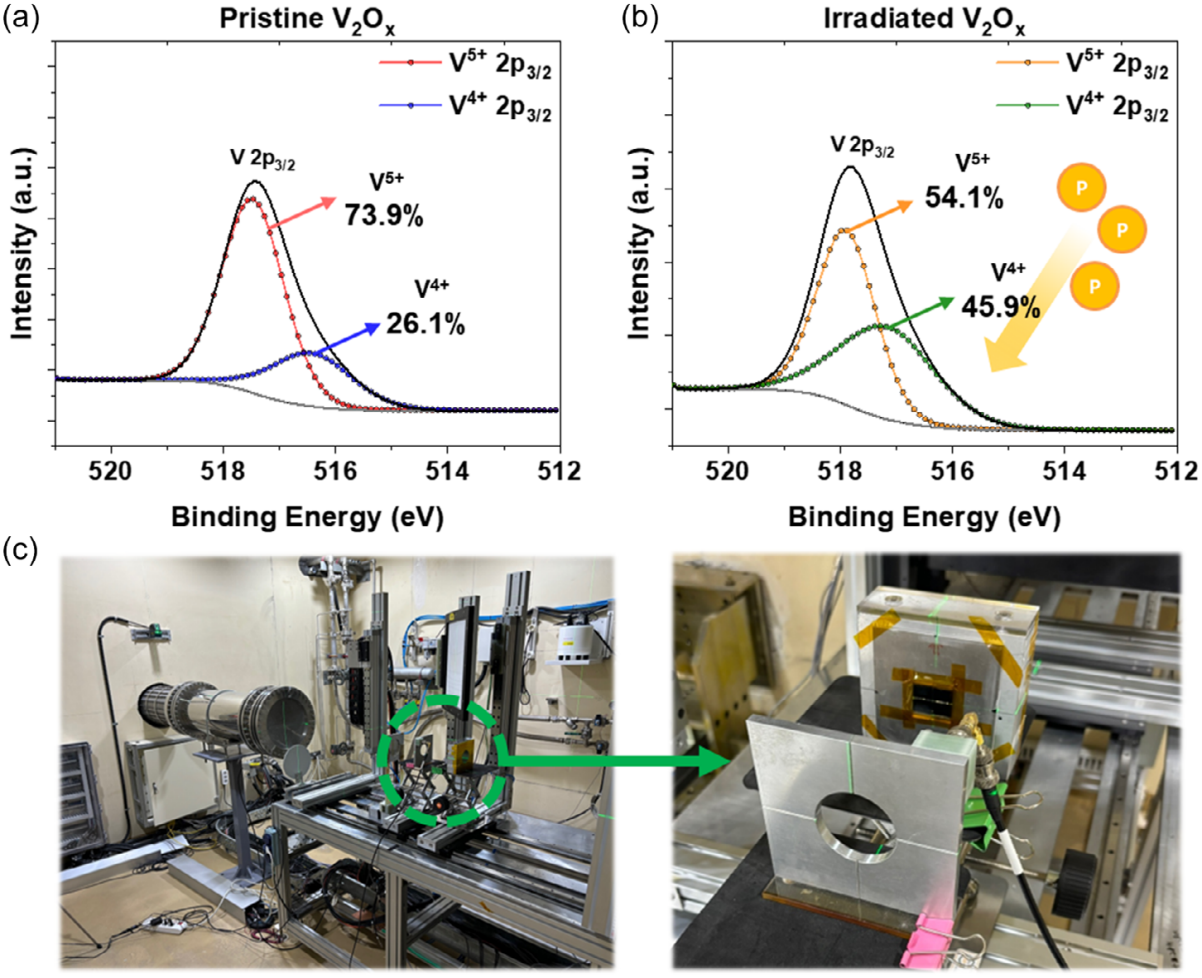
XPS analysis of V 2p core levels and proton beam system. V 2p spectra of V_2_O*_x_* films measured (a) before irradiation and (b) after 15 MeV proton irradiation at fluences of 10^12^ p/cm^2^ (approximately equivalent to 35–40 years of accumulated dose in LEO). (c) Photograph of the proton beam irradiation system and sample mounting configuration.

To clarify the origin of oxygen deficiency, XPS was performed on thermally evaporated V_2_O*_x_* films. In Figure 2a, XPS analysis of the thermally evaporated V_2_O*_x_* films reveals pronounced oxygen deficiency and mixed vanadium oxidation states. The V 2p_3/2_ peak can be deconvoluted into two distinct contributions, V^5+^ at a binding energy of 517.4 eV (73.9%) and V^4+^ at 516.4 eV (26.1%). Based on the deconvolutions, the V^4+^/V^5+^ ratio of ≈26.1:76.3 directly indicates deviation from stoichiometric V_2_O_5_. Previous studies have shown that low‐pressure, oxygen‐poor environments during thermal evaporation favor partial reduction through incomplete oxygen incorporation, leading to the formation of substoichiometric vanadium oxide phases [22, 30]. Based on charge neutrality considerations, the measured V^4+^ fraction corresponds to a substoichiometric composition of V_2_O_5‐*x*_ with *x* ≈ 0.26 (≈V_2_O_4.74_), indicating the presence of a significant concentration of oxygen vacancies. This corresponds to an oxygen vacancy concentration of ≈5.2% relative to the anion sublattice (V_O▪▪_ defects).

Consistent with the V 2p analysis, the O 1s spectrum (Figure S3) can be deconvoluted into lattice oxygen bonded to vanadium (O_L_) at 530.4 eV (73.6%) and defect‐related oxygen (O_D_) at 531.4 eV (26.4%). The O_D_ component is attributed to oxygen atoms adjacent to oxygen vacancies or undercoordinated oxygen species at the surface and near surface regions of the evaporated films. Together, the V 2p and O 1s analyses provide direct evidence that thermal evaporation intrinsically produces oxygen‐deficient V_2_O*_x_* films without the need for additional postdeposition treatments. In V_2_O*_x_*‐based oxides, oxygen vacancies introduce shallow donor states that increase carrier concentration and enhance electrical conductivity while preserving wide bandgap optical transparency [31, 32, 33]. The presence of V^4+^ species associated with oxygen vacancy compensation generates localized electronic states within the bandgap, which can act as unintentional doping sources and influence interfacial charge extraction [34].

To evaluate the changes of V_2_O*_x_* after irradiation, proton irradiation was conducted and the result of XPS V 2p core level is shown in Figure 2b. V_2_O*_x_* layer deposited on the substrates was exposed under the proton radiation condition of 15 MeV with a fluence of 10^12^ p/cm^2^ as shown in Figure 2c. A fluence of 10^12^ p/cm^2^ at 15 MeV corresponds to 35–40 years of accumulated LEO exposure [35]. XPS analysis reveals significant modifications to the V_2_O*_x_* composition with increasing proton fluence, as evidenced by an increase in the V^4+^ content from 26.1% to 45.9% (Figure 2b), indicating that proton‐induced point defects progressively create additional reducing conditions within the V_2_O*_x_* film. Such composition changes are consistent with secondary defect creation in metal oxides under energetic ion bombardment, where inelastic collisions can preferentially displace oxygen atoms due to their lower atomic mass and binding energy compared with vanadium [36, 37, 38].

### 3.2 V_2_O*_x_* HTL for Radiation‐Hardened Perovskite Solar Cells

**FIGURE 3:**
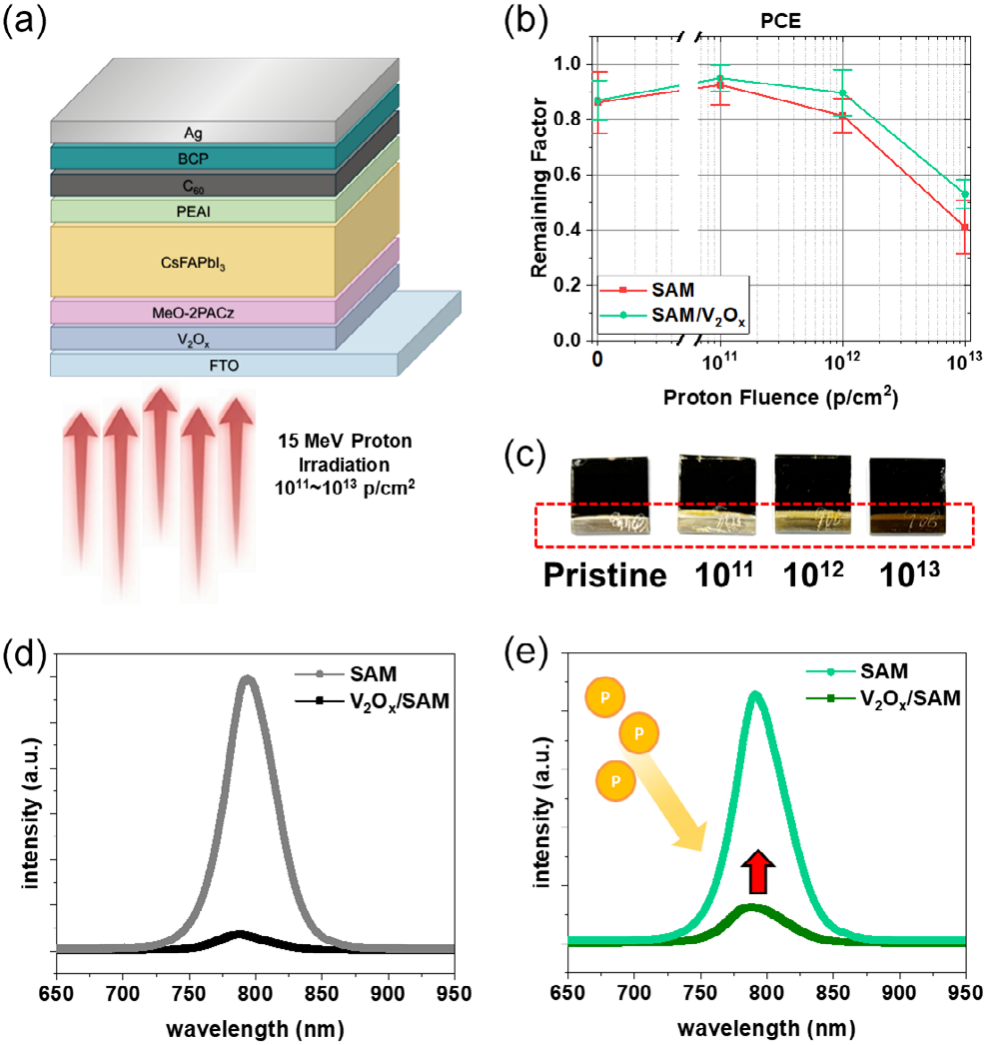
Photovoltaic performance. (a) Schematic device architectures of radiation‐hardened perovskite solar cells employing a V_2_O*_x_*/SAM bi‐HTL, (b) remaining PCE factor of SAM‐only control devices and V_2_O*_x_*/SAM bi‐HTL devices after 15 MeV proton irradiation at fluences of 0 (pristine), 10^11^, 10^12^, and 10^13^ p/cm^2^, (c) photoimages of devices after irradiation with various fluences, and steady‐state PL quenching of SAM‐only samples and V_2_O*_x_*/SAM bi‐HTLs (d) before and (e) after 15 MeV irradiation condition at fluence of 10^12^ p/cm^2^.

Perovskite solar cells with Cs_0.05_FA_0.95_PbI_3_ (hereafter referred to as CsFAPbI_3_), structured as shown in Figure 3a, incorporating thermally evaporated V_2_O*_x_* and SAM bi‐HTLs demonstrated strong baseline photovoltaic performance with average power conversion efficiency (PCE) of 23.2% ± 0.3% under AM1.5G illumination (100 mW/cm^2^) as shown in Figure S4. Notably, forward–reverse IV hysteresis was negligible (< 1%), indicating suppressed ion migration at the inorganic oxide–perovskite interface. The obtained photovoltaic parameters across at least 20 independent devices are shown in Table S1. Notably, devices employing the V_2_O*_x_*/SAM bi‐HTL perovskite devices exhibited systematically enhanced photovoltaic performance compared to control devices using a SAM‐only HTL perovskite devices, highlighting the beneficial role of the inorganic V_2_O*_x_* overlayer. Short‐circuit current density (*J*_sc_) of V_2_O*_x_*/SAM devices averaged 25.1 ± 0.33 mA/cm^2^, compared to that of SAM‐only devices (24.8 ± 0.41 mA/cm^2^), reflecting excellent electrical performances and efficient light harvesting in the perovskite. The external quantum efficiency spectra (Figure S5) show nearly identical onset behavior for both devices. Open‐circuit voltage (*V*_oc_) of 1.12 ± 0.03 V indicates favorable band alignment at the V_2_O*_x_*/SAM/perovskite interface, while fill factors of 79.5% ± 1.2% suggest good charge collection and minimal interfacial recombination.

To confirm benefits of V_2_O*_x_* in terms of radiation hardness, perovskite devices were exposed to 15 MeV proton radiation with fluences of 10^11^–10^13^ p/cm^2^ (Figure 3a). The radiation incidence direction is set from the FTO side to evaluate the role of the V_2_O*_x_* layer and to reflect LEO missions in which single‐junction perovskite solar cells operate as a space photovoltaics. In Figure 3b, both devices retained around 0.9 of the initial PCE up to 10^11^ p/cm^2^, indicating that limited performance loss in the low‐fluence case. As the dose increases up to 10^12^ p/cm^2^, the SAM‐only devices exhibit a more reduction in performance, whereas the V_2_O*_x_*/SAM devices exhibit a higher performance, suggesting that the V_2_O*x* interlayer mitigates radiation‐induced degradation. Continuously, at 10^13^ p/cm^2^, substantial degradation is observed for both devices although the V_2_O*_x_*/SAM devices consistently show better performances, implying that partial protection rather than complete suppression of damage. The main reason for performance drops at the highest fluence (10^13^ p/cm^2^) is attributed to radiation‐induced substrate darkening (Figure 3c), consistent with previous reports [39, 40]. The substrate darkening phenomena limit optical transmission and the light harvesting of the perovskite layer, leading to reduced light absorption and a pronounced decrease in *J*_sc_ (Figure S6a). Additionally, we confirmed that *V*_oc_ and FF of V_2_O*_x_*/SAM devices remained higher than that of SAM‐only devices in Figures S6b–c, indicating that V_2_O*_x_* interlayer suppresses ionizing radiation damage.

To further evaluate the role of V_2_O*_x_* interlayer, PL was conducted on CsFAPbI_3_ perovskite films on V_2_O*_x_*/SAM bi‐HTL and on SAM‐only HTL samples. After 15 MeV proton irradiation, steady‐state PL measurements reveal an impact of interfacial charge extraction in devices employing a V_2_O*_x_*/SAM bi‐HTL. At pristine condition (Figure 3d) and 10^11^ p/cm^2^ (Figure S7a), the bi‐HTL exhibits stronger PL quenching, compared to the SAM‐only samples, indicating enhanced charge extraction and suppressed nonradiative recombination, which can be attributed to a tunneling‐assisted transport effect introduced by the V_2_O*_x_* layer. However, as the fluence increases up to 10^12^ p/cm^2^, the charge extraction capability of the bi‐HTL progressively diminishes as shown in Figure 3e and Figure S7b, presumably because V_2_O*_x_* undergoes irradiation‐induced reduction and interacts with the carbazole‐based SAM, leading to a loss of *π*‐conjugation [41, 42, 43].

**FIGURE 4:**
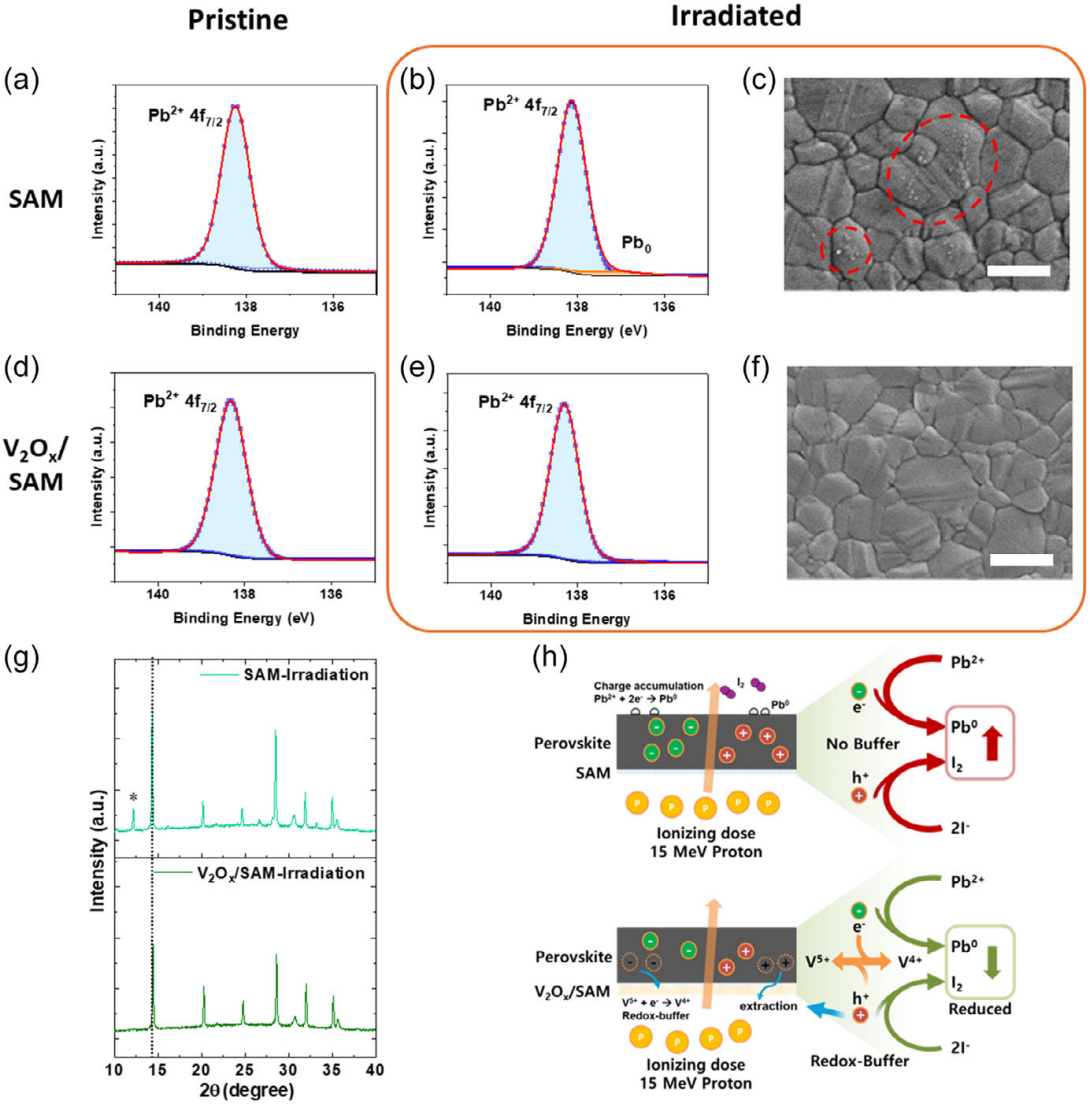
Suppressed Pb^0^ formation after irradiation. Pb 4f XPS spectra of SAM‐only samples (a) before and (b) after irradiation, together with (c) a top‐view SEM image after irradiation. Pb 4f XPS spectra of V_2_O*_x_*/SAM samples (d) before and (e) after irradiation, together with (f) a top‐view SEM image after irradiation, (g) XRD patterns of irradiated samples for comparison in which the * marker and dash line in the image indicate uncoordinated Pb^2+^ and *α*‐phase of perovskite, and (h) schematic illustration of the proposed redox‐active interactions between V_2_O*_x_* and perovskite‐derived lead (Pb) and halide (I) species.

Proton irradiation induces chemical‐state changes in the perovskite layer, as revealed by comparative XPS analysis of the Pb 4f for devices employing the V_2_O*_x_*/SAM bi‐HTL, compared with the SAM‐only HTL. In the SAM‐only samples, a metallic Pb component (Pb^0^), which is not shown in the pristine reference (Figure 4a), becomes increasingly pronounced with increasing proton fluence to 10^12^ p/cm^2^ (Figure 4b), indicating progressive accumulation of reduced Pb species under irradiation. Meanwhile, V_2_O*_x_*/SAM samples, as shown in Figure 4d,e, have no signal of metallic Pb^0^ peak before and after irradiation. Metallic Pb^0^ peak of SAM‐only samples became greater at fluence of 10^13^ p/cm^2^ (Figure S8a), while V_2_O*_x_*/SAM samples did not show metallic Pb^0^ even at the highest fluence we tested as shown in Figure S8b. This trend can be attributed to halide‐loss process in which iodide (I^−^) depletion generates iodide vacancies (V_I_) and Pb‐rich, uncoordinated Pb^2+^ sites, thereby accelerating Pb—I bond breakage and forming metallic Pb at/near the surface [44, 45, 46]. XRD result of irradiated samples in Figure 4g confirms the presence of uncoordinated Pb^2+^ in SAM‐only samples after irradiation, which is consistent with XPS results. It is also confirmed that the halide (I)‐to‐lead (Pb) ratio for the SAM‐only samples (1.82) is lower than that for the V_2_O*_x_*/SAM samples (2.30) at a fluence of 10^13^ p/cm^2^, suggesting relatively greater iodide (I^−^) depletion in SAM‐only samples (Table S2 and Figure S9). Furthermore, top‐view SEM analysis examines radiation‐induced morphological changes in perovskite films with the designed HTLs. In Figure 4c, the SAM‐only samples exhibit abnormal pimple‐like points on the perovskite grains after irradiation, whereas such points are not observed in the V_2_O*_x_*/SAM samples as shown in Figure 4f, indicating that the V_2_O*_x_* interlayer mitigates surface degradation. These results indicate that the incorporation of V_2_O*_x_* at the hole‐transporting contact suppresses irradiation‐induced Pb reduction at the perovskite surface.

The absence of Pb^0^ in the V_2_O*_x_*/SAM devices is consistent with an interfacial stabilization mechanism in which mixed‐valence V_2_O*_x_* buffers irradiation‐driven charge imbalance and reduces the probability of Pb reduction and halide volatilization pathways, as shown in Figure 4h. Under TID‐dominated irradiation, continuous generation of nonequilibrium carriers and enhanced charge trapping near interfaces can promote electron‐driven reduction unless excess electrons are efficiently extracted or buffered. In SAM‐only devices, such electron accumulation can drive the reduction of Pb^2+^ to Pb^0^ [47, 48], as indicated by Equation (1).

(1) $${\mathrm{Pb}}^{2+}+2e^{-}\to {\mathrm{Pb}}^{0}$$

By introducing a V_2_O*_x_* containing bilayer HTL, mixed‐valence vanadium sites can provide a competing electron‐accepting pathway, schematically corresponding to partial reduction of V^5+^ toward V^4+^, thereby lowering the effective electron chemical potential at the interface [49, 50].

This competition can suppress the net flux of electrons into Pb^2+^ reduction, thereby mitigating Pb^0^ formation even with fluence increases. In parallel, irradiation‐induced enrichment of V^4+^/oxygen vacancy‐related states may improve hole extraction at the interface of HTL/perovskite, therefore decreasing hole retention time and interface accumulation in the perovskite and thus indirectly suppressing iodide oxidation and related halide vacancy formation that contribute to iodine‐containing volatile species.

(2) $$2I^{-}+2h^{+}\to I_{2}$$

Thermodynamically, the I_2_/I^−^ couple has a standard reduction potential of ≈+ 0.535V versus standard hydrogen electrode (SHE), whereas the aqueous V^5+^/V^4+^ couple commonly used for comparison, VO_2_^+^/VO^2+^, has a standard potential of ≈+ 1.00 V versus SHE [47, 48, 49]. From this thermodynamic perspective, iodide oxidation to I_2_ requires a lower oxidizing driving force than oxidation of V^4+^ to V^5+^, suggesting that iodide oxidation is thermodynamically more favorable. Therefore, suppression of iodine‐containing volatile species in the V_2_O*_x_*/SAM bi‐HTL devices is more conservatively interpreted as an indirect consequence of reduced interfacial hole accumulation and faster hole extraction, rather than as dominant direct hole consumption by V_2_O*_x_* via a chemical oxidation step. Nevertheless, under steady carrier generation and interfacial charge exchange, the mixed‐valence V_2_O*_x_* layer can still be described as a redox‐cycling interfacial buffer, in which reversible V^5+^/V^4+^ conversion helps dissipate transient charge imbalance while maintaining hole extraction functionality [49].

Overall, the Pb 4f XPS results support that incorporating V_2_O*_x_* at the hole‐selective contact enhances the resistance of the perovskite/HTL interface against proton‐induced chemical reduction pathways. This improvement can not only mitigate defect‐assisted electron accumulation but also limit the halide‐loss scenario (V_I_ formation → undercoordinated Pb^2+^ → Pb^0^) in space, which can be relevant to reducing halide outgassing and improving space reliability of radiation‐hardened perovskite solar cells.

## 4 Conclusions

Thermally evaporated V_2_O*_x_* forms an amorphous, oxygen‐deficient HTL with mixed V^5+^/V^4+^ states and favorable energetics for perovskite solar cells, enabling efficient hole‐selective contact when combined with a SAM. Proton irradiation studies reveal that V_2_O*_x_*/SAM bi‐HTL enhances charge extraction at lower fluences (up to 10^12^ p/cm^2^), whereas higher fluences progressively weaken this advantage, indicating an interface‐dominated degradation mechanism rather than bulk perovskite structural failure, confirmed by XPS. Additional XPS analysis shows that proton irradiation induces further reduction of V_2_O*_x_*, accompanied by oxygen vacancy formation and an increased V^4+^ fraction, consistent with a redox‐active interface. Notably, Pb core‐level spectra show a clear contrast between bi‐HTL devices and SAM‐only devices, with SAM‐only devices exhibiting irradiation‐induced metallic Pb (Pb^0^) formation, which is possibly associated with halide‐loss‐driven degradation such as iodine volatilization. In contrast, V_2_O*_x_*/SAM bi‐HTL devices suppress the formation of Pb^0^ and limit halide volatilization across the investigated irradiation conditions. These results establish metal oxide/SAM interfacial engineering as an effective strategy to stabilize perovskite interfaces against proton‐induced chemical reduction. Beyond conventional passivation strategies, the incorporation of a redox‐active interfacial couple can buffer irradiation‐induced charge and mitigate irreversible chemical changes at the perovskite interfaces. This concept offers a practical pathway toward radiation‐tolerant, lightweight perovskite photovoltaics for space applications.

## Supporting information

Supplementary Material
