# Supplementary material for: Radiation‐Hardened Perovskite Solar Cells Enabled by Redox‐Active V2O x Hole Transport Layer for Space Applications
Source: Small Sci. 2026 May 28;6(6):e70304. doi: 10.1002/smsc.70304 (PMC13248843; doi:10.1002/smsc.70304)
Supplement: Supplementary file 1 — Supplementary Material [file SMSC-6-e70304-s001.pdf]

small science

Radiation-Hardened Perovskite Solar Cells Enabled by Redox-Active V_2O_x Hole Transport Layer for Space Applications

EQ Han[a], Su-Ho Ahn[a], Eunyoung Choi[a], Su-Min Lee[a], Jaeho Lee[b], Bo Wei Zhang[b], Miaoqiang Lyu[b], Jung-Ho Yun[a]*

[a] *Air & Environment Energy Nexus (A2EN) Laboratory, Department of Environmental Science and Engineering, College of Engineering, Kyung Hee University, Gyeonggi-do 17104, Republic of Korea*
[b] *Nanomaterials Centre, School of Chemical Engineering, Australian Institute for Bioengineering and Nanotechnology, The University of Queensland, Brisbane, QLD 4072, Australia*

* Corresponding authors.
E-mail address: jungho.yun@khu.ac.kr (J.-H. Yun)

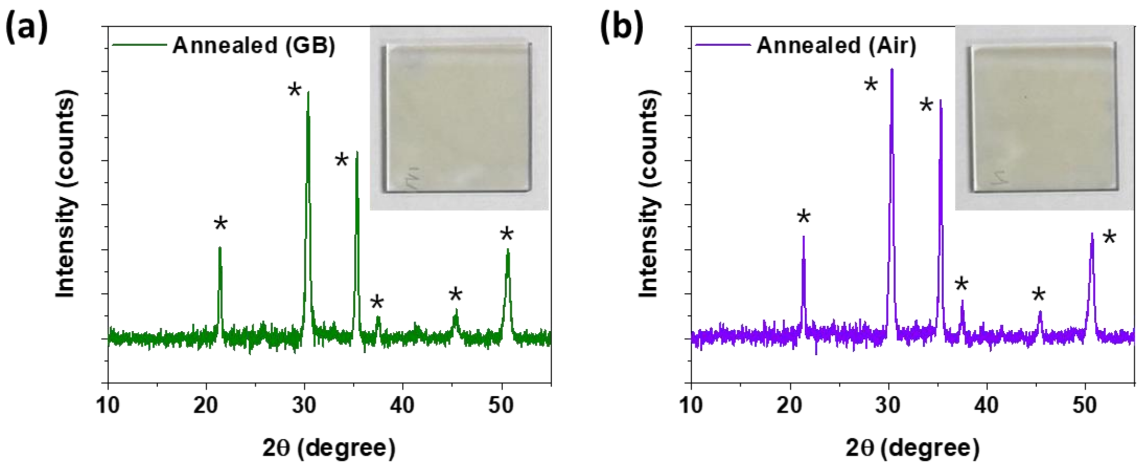

Figure S1. Structural stability of V_2O_x after post-annealing. XRD patterns of V_2O_x films after post-annealing at 150 °C for 5 min (a) in an N_2-filled glovebox (GB) and (b) in ambient conditions (Air), confirming retention of the amorphous phase in both cases. * markers in the figures indicate ITO peaks.

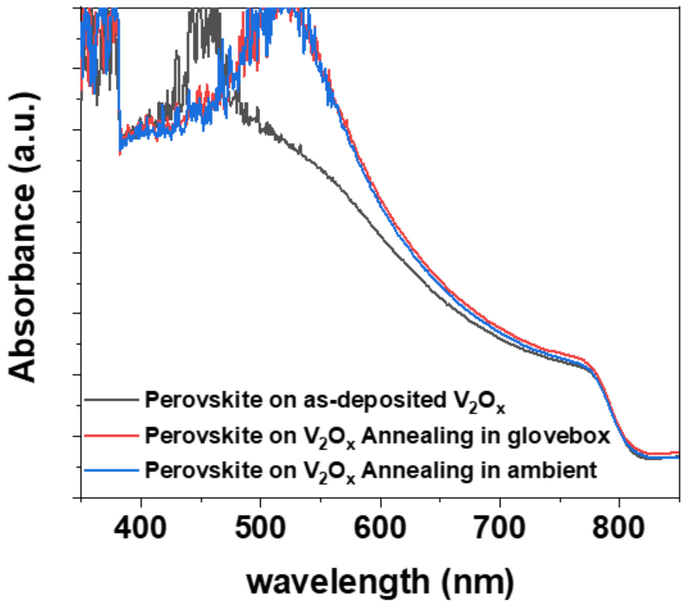

Figure S2. UV–visible absorption spectra. Absorbance spectra of perovskite films deposited on thermally evaporated V_2O_x layers under different post-treatments: as-deposited V_2O_x and V_2O_x post-annealed at 150 °C for 5 min in an N_2-filled glovebox or in ambient air.

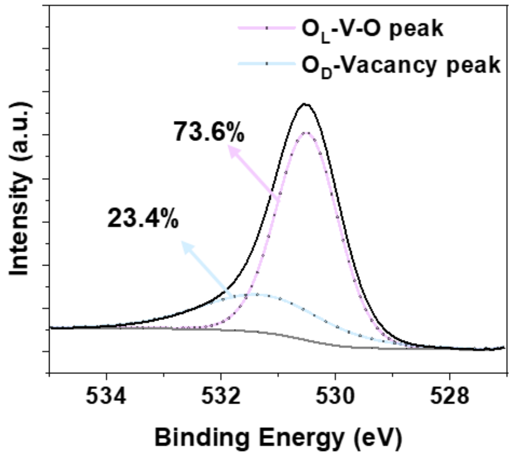

Figure S3. XPS analysis of oxygen bonding states. XPS O 1s core-level spectra of as-deposited V_2O_x films.

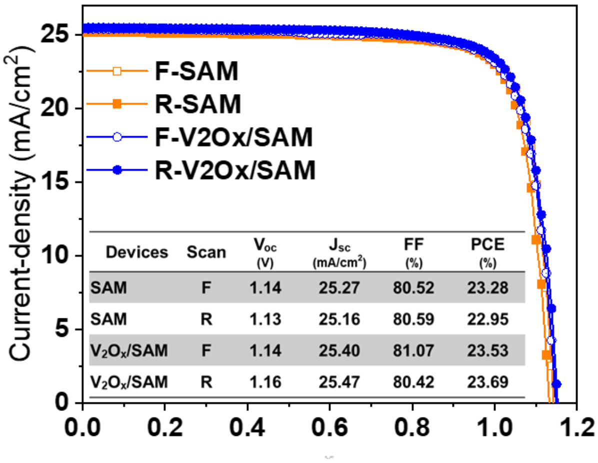

Figure S4. Solar cell performances. *J*-*V* performances of the champion solar cells for SAM-only and V_2O_x/SAM perovskite solar cell devices. Both devices show negligible hysteresis.

small science

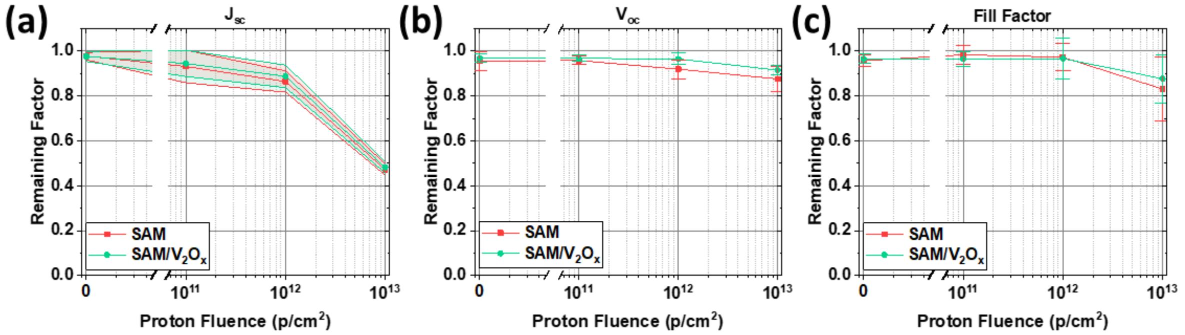

Figure S5. Remaining factors of photovoltaic parameters after irradiation of 15 MeV. (a) J_{sc}, (b) V_{oc}, and (c) fill factor.

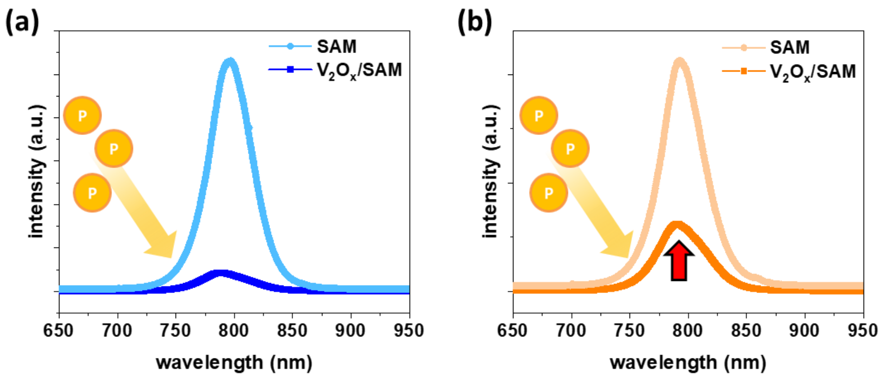

Figure S6. Steady-state photoluminescence after 15 MeV proton irradiation. (a) 10^{11} and (b) 10^{13} p/cm^2 fluences exposed.

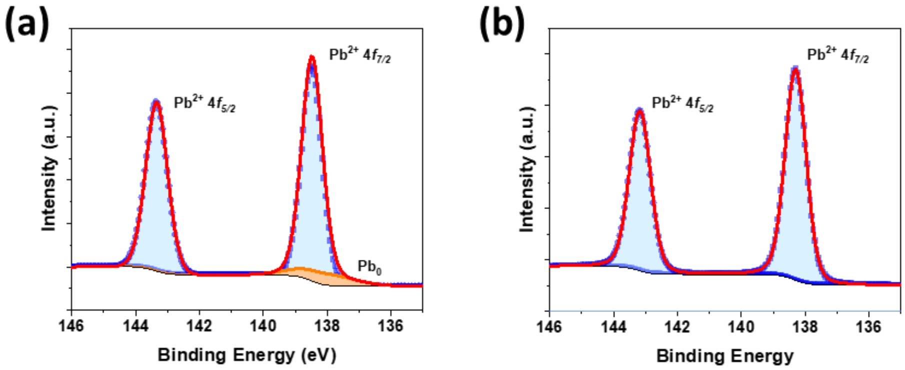

Figure S7. Pb 4f spectra obtained by XPS after 10^{13} p/cm^2 dose of proton. (a) SAM-only and (b) SAM/V_2O_x samples.

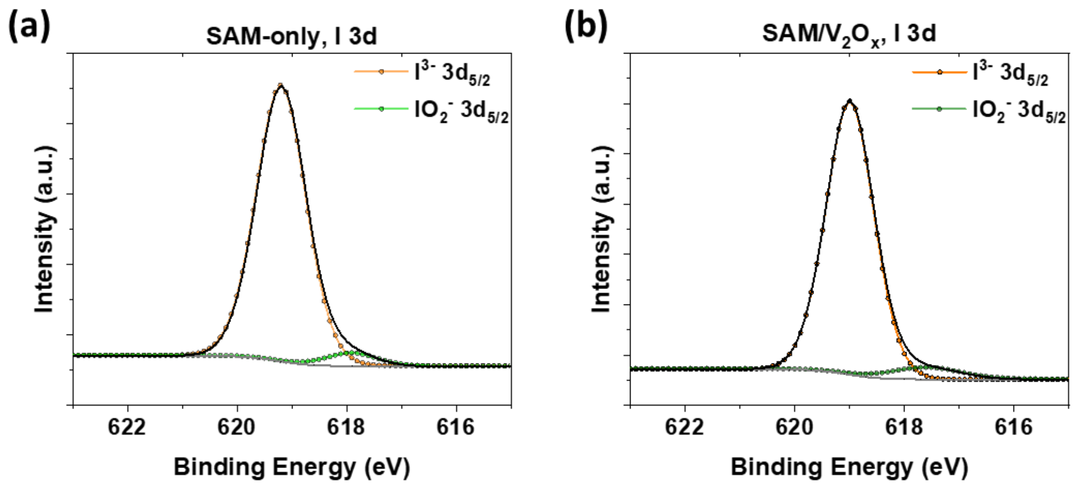

Figure S8. Halide retention after irradiation. XPS I 3d core-level spectra of (a) SAM-only sample and (b) V_2O_x/SAM, measured at fluences of 10^{12} p/cm^2.

Table S1. Photovoltaic performance summary. Summary of photovoltaic parameters for control (SAM-only HTL) and designed (V_2O_x/SAM bilayer HTL) devices (n = 20 devices for each condition).

Devices	V_{oc} (V)	J_{sc} (mA/cm^2)	FF (%)	PCE (%)
**SAM**	1.10 ± 0.02	24.80 ± 0.41	79.3 ± 1.3	22.70 ± 0.30
**V_2O_x/SAM**	1.12 ± 0.03	25.10 ± 0.33	79.5 ± 1.2	23.20 ± 0.29

small science

Table S2. Summary of the halide (I)-to-lead (Pb) ratio obtained from XPS. Peak areas were extracted from the fitted spectra and the I/Pb ratio after irradiation of 10^{12} and 10^{13} p/cm^2 was calculated using Relative Sensitivity Factor (R.S.F)-corrected intensities, considering only the I $3d_{5/2}$ and Pb $4f_{7/2}$ components.

		**I $3d_{5/2}$ RSF$_I$=33.64**		**Pb $4f_{7/2}$ RSF$_{Pb}$=22.74**		**I/Pb ratio**
**Fluence p/cm^2**	**Devices**	**I^{3-}**	**IO_2^-**	**Pb^{2-}**	**Pb^0**	**[(I^{3-} + IO_2^-)/R.S.F$_I$] / [(Pb2- + Pb^0)/R.S.F$_{Pb}$]**
**10^{12}**	SAM-only	125947.6	29838	39483.6	7919.3	2.22
**10^{12}**	SAM/V_2O_x	103530.8	27654.5	34954.2	1217.5	2.45
**10^{13}**	SAM-only	75534.9	20029.2	26845.1	8498.2	1.82
**10^{13}**	SAM/V_2O_x	84466.4	19587	29418.7	1112.1	2.30
